# De novo Transcriptome of the Non-saxitoxin Producing *Alexandrium tamutum* Reveals New Insights on Harmful Dinoflagellates

**DOI:** 10.3390/md18080386

**Published:** 2020-07-24

**Authors:** Giorgio Maria Vingiani, Dārta Štālberga, Pasquale De Luca, Adrianna Ianora, Daniele De Luca, Chiara Lauritano

**Affiliations:** 1 Marine Biotechnology Department, Stazione Zoologica Anton Dohrn, Villa Comunale, CAP80121 Napoli, Italy; giorgio.vingiani@szn.it (G.M.V.); adrianna.ianora@szn.it (A.I.); 2 Faculty of Medicine and Health Sciences, Linköping University, 58183 Linköping, Sweden; stalberga.darta@gmail.com; 3 Research Infrastructure for Marine Biological Resources Department, Stazione Zoologica Anton Dohrn, Villa Comunale, CAP80121 Napoli, Italy; pasquale.deluca@szn.it; 4 Department of Humanities, Università degli Studi Suor Orsola Benincasa, CAP80135 Naples, Italy; daniele.deluca088@gmail.com

**Keywords:** dinoflagellates, *Alexandrium tamutum*, transcriptomics, toxin producing enzymes, harmful algal blooms

## Abstract

Many dinoflagellates species, especially of the *Alexandrium* genus, produce a series of toxins with tremendous impacts on human and environmental health, and tourism economies. *Alexandrium tamutum* was discovered for the first time in the Gulf of Naples, and it is not known to produce saxitoxins. However, a clone of *A. tamutum* from the same Gulf showed copepod reproduction impairment and antiproliferative activity. In this study, the full transcriptome of the dinoflagellate *A. tamutum* is presented in both control and phosphate starvation conditions. RNA-seq approach was used for in silico identification of transcripts that can be involved in the synthesis of toxic compounds. Phosphate starvation was selected because it is known to induce toxin production for other *Alexandrium* spp. Results showed the presence of three transcripts related to saxitoxin synthesis (sxtA, sxtG and sxtU), and others potentially related to the synthesis of additional toxic compounds (e.g., 44 transcripts annotated as “polyketide synthase”). These data suggest that even if this *A. tamutum* clone does not produce saxitoxins, it has the potential to produce toxic metabolites, in line with the previously observed activity. These data give new insights into toxic microalgae, toxin production and their potential applications for the treatment of human pathologies.

## 1. Introduction

Harmful algal blooms (HABs) are intense algal proliferations that can profoundly disrupt the habitat where they occur via the production of high biomass accumulation, which causes oxygen depletion and the production of high amounts of toxins [1,2]. HABs induce detrimental effects on marine organisms, humans, environments and economies [1,3]. They are often associated to toxin-producing freshwater cyanobacteria and marine dinoflagellates [4]. Dinoflagellates, together with diatoms, are the main primary producers in aquatic ecosystems. *Alexandrium* spp. is one of the most common marine dinoflagellates known to be responsible for seasonal HABs; there are more than 40 species, half of which are known to produce toxins [5]. Microalgae are known to produce a series of compounds, generally derived from secondary metabolism, known to regulate and control algal biology, species succession, communication and competition, and predator deterrence [6]. The main toxins produced by *Alexandrium* spp. are saxitoxins and their analogues (57 natural saxitoxin analogues are known to date [4]), and neurotoxic alkaloids responsible for paralytic shellfish poisoning (PSP), such as neosaxitoxin, gonyautoxin, spirolides and goniodomines [5].

HABs are known to be responsible for shellfish-borne toxicity, fish kills, as well as human intoxications (generally due to bioaccumulation by filter-feeding bivalves and fish, and subsequent transfer through the food web), and even death [5,7,8]. Saxitoxin and its analogues have the ability to bind to voltage-gated sodium, calcium and potassium channels, and lead to death via respiratory paralysis [9]. Algal toxins have been reported to cause 50,000–500,000 human intoxications per year, with an overall global mortality rate of 1.5% [9]. The number of human deaths has stimulated studies on dinoflagellate toxins and their synthesis, which is still not clear, their toxicity on marine organisms and humans, as well as the development of new technologies for toxin detection and HAB monitoring [10]. Toxicity is highly variable between different clones of the same species [11]. This is the case, for example, of *A. minutum* sampled along the Irish coast, where clones collected on the southern coast were toxic, while clones collected on the western coast were not [12].

*Alexandrium tamutum* was described for the first time by Montresor and colleagues [13] from the Gulf of Naples, Mediterranean Sea. Their clone was also chemically analysed, and known toxins were not detected, classifying the strain as non-toxic. In addition to the Mediterranean Sea, *A. tamutum* was also identified in Scottish [14], Malaysian [15] and Chinese waters [16]. The Scottish strain was found negative for PSP toxins as well. Ianora et al. [6] isolated from the Gulf of Naples another clone of *A. tamutum*, named at that time as *A. tamarense* FE107, whose ingestion by the copepod *Temora stylifera* induced reproduction impairment via a dramatic reduction in copepod egg production and hatching success, with egg viability dropping to 0% after 24 h of feeding. Chemical analyses were also performed and toxins were not detected (HPLC analysis excluded a cytotoxicity related to saxitoxins). The FE107 clone was successively characterized by 18S sequencing genotyping and identified as *A. tamutum* [17]. Lauritano and co-workers also tested the anti-proliferative activity of *A. tamutum* FE107 on human hepatocellular liver carcinoma (HepG2, ATCC HB-8065^TM^) after 24 h exposure, as well as on normal human lung fibroblast (MRC-5, ATCC CCL-171^TM^)), and human melanoma cells (A2058, ATCC CRL-11147^TM^) after 72 h exposure. Results showed that there was a strong anti-proliferative activity on MRC-5 cells incubated with 50 μg/mL *A. tamutum* FE107 extract, and on A2058 cells when incubated with 100, 50, 25, 12.5 and 10 μg/mL *A. tamutum* FE107 extract. On the contrary, 24 h FE107 exposure did not show any effect on HepG2 cells [17]. 

Considering that *A. tamutum* showed toxic effects on copepod reproduction and anti-proliferative activity on human cells, it was hypothesized that it may produce some toxic compounds other than saxitoxins which were not detected by the chemical analyses. In this study, the full transcriptome of the dinoflagellate *A. tamutum* FE107 is reported and in silico identification of transcripts that can be involved in the synthesis of toxic compounds is performed. Until now, various studies have focused on characterizing the saxitoxin biosynthetic pathway [18,19], however, currently available data have not fully identified it. Different studies have used “omics” technologies to shed light on natural product biosynthesis. However, due to their huge genome sizes (3–245 Gbp [4]), molecular resources for dinoflagellates are still scarce [20], and mainly related to transcriptomic data. 

Based on bioinformatic predictions merged from different studies, the putative pathway for saxitoxin biosynthesis in cyanobacteria begins with sxtA (with four catalytic domains named sxtA1, sxtA2, sxtA3 and sxtA4), followed by sxtG, sxtB, sxtC, sxtD, sxtS, sxtU, sxtH/T, sxtV and sxtW [4]). Saxitoxin analogues are then synthesized starting from saxitoxin to sxtI, sxtJ, sxtK, sxtL, sxtN, sxtO and sxtX. In addition, there are a series of regulatory genes that allow the regulation of saxitoxin production in response to environmental stimuli and/or modification of the production at the post-transcriptional level [4]. In the transcriptome of various saxitoxin-producing dinoflagellates, several of these genes have also been identified [21,22,23]. Previous studies have shown that not all *Alexandrium* spp. have the same *sxt* genes, and differences were mainly assigned comparing toxic versus non-toxic strains. For example, Hackett [19] showed that the C-terminal region of sxtA (referring to the cyanobacterial domain sxtA4) was exclusively found in saxitoxin-producing strains. Zhang et al. [24] found the same domain in the genome of non-toxic mutants of *Alexandrium catenella,* but it was not found expressed (by both RNA-seq and qPCR analyses). These data suggest that sxtA4 gene is present in the genome but it is not transcribed in the studied conditions. More ambiguous evidence appeared regarding the sxtG gene. This gene, previously considered another marker of saxitoxin-producing strains, was also found in three non-saxitoxin-producing *Alexandrium* strains (even if the absence in such transcripts of a stop codon could cause its non-functionality) [23]. Moreover, as previously mentioned, a few *Alexandrium* species also showed the production of other toxins, whose biosynthetic pathways are less known, precisely spirolides (SPXs) (e.g., *Alexandrium ostenfeldii*, [25]), gymnodimines (GYMs) (e.g., *Alexandrium ostenfeldii* [26,27]) and goniodomins (e.g., *Alexandrium hiranoi* [28,29]). Finally, some cases were reported of strains capable of producing an unidentified ichthyotoxin, but not PSP [30], showing that the toxicological profile of *Alexandrium* spp. still needs to be fully explored.

Considering that *Alexandrium* spp. have been shown to increase the production of toxins under phosphate starvation (P-starvation) culturing condition [31,32,33], the transcriptome sequencing of *A. tamutum* cultured under phosphate starvation has also been performed in this study in order to stimulate the transcription of *sxt* or toxin-related genes. Finally, differential expression analysis between the two conditions (control and P-starvation) was also performed in order to identify (1) *sxt* genes, (2) metabolic changes induced by P-starvation, and (3) possible other sequences involved in toxin synthesis, or compounds of biotechnological interest. 

## 2. Results and Discussion

### 2.1. Transcriptome Sequencing and De Novo Assembly

RNA-sequencing (RNA-seq) from six samples, three cultured in control culturing condition and three in P-starvation, yielded 164,128,287 and 62,180,610 total raw and normalized fragments, respectively. Normalised RNA-seq reads were assembled with a de novo approach, because no available reference genome of *A. tamutum* was available, obtaining a raw assembly of 293,633 transcripts grouped into 220,519 genes. The mean GC content was 65.23%. The average and the median contig length were 776.87 bp and 472 bp, respectively. The N50 was 1202 bp. Controls were performed on the raw transcriptome assembly in order to check for its quality. Transcripts were translated into proteins with Transdecoder, obtaining a total of 182,156 protein sequences (minimum length 50aa). Among these, 26,506 (14.55%) were complete (with a methionine and a stop codon), 12,782 (7.01%) started with a methionine, but lacked a stop codon, 72,875 (40%) only had a stop codon, and 69,993 (38.42%) did not start with a methionine and did not have a stop codon. Protein sequences were blasted against two datasets of the core eukaryotic genes [34] to verify completeness of the assembly, including 248 and 458 protein sequences; respectively 248 out of 248 (100%) and 458 out of 458 (100%) could be detected in the assembly. The assembly protein length was also compared to the length of the core eukaryotic genes. About 460 of the proteins covered more than 90% of the length of the corresponding core eukaryotic proteins and 663 covered more than 80% of the length of the corresponding core eukaryotic protein. Considering that RNA-seq can suffer from the contamination of organisms that are not the target of the sequencing, transcript sequences were blasted against the NCBI database of bacteria and Archaea in order to remove possible contaminations. By this procedure, 3156 transcripts were detected and removed. The distribution of the GC content was also analysed in the dataset; it followed a normal distribution with a mean value of 65.1% and a standard deviation of 3.48. By using a *z*-test, 60,299 sequences were identified to have a GC content significantly different from the observed mean (*p* < 0.01). These sequences were blasted against the NCBI (NR) database to look for contaminants, and 803 sequences were removed due to matching with bacterial or metazoan sequences. The obtained filtered assembly was composed by 28,9674 transcripts, grouped into 21,7725 genes. The mean GC content was 65.31%. The average and the median contig lengths were 780.16 bp and 475 bp, respectively, and the N50 was 1206 bp.

### 2.2. Functional Annotation

The sequences of the assembled transcripts were translated into proteins by using Transdecoder (minimum length of 50aa). If multiple translations were possible, the longest complete ORF was kept, and if a complete ORF was not detected, the longest sequence was maintained. The sequences were also analysed for the presence of repetitive elements (i.e., DNA transposons, retroelements, satellites, rRNA, etc.) with Repeat Masker (version open-4.0.5, [35]) that were then removed. In order to associate a function to the assembled transcripts, Blast2GO software was used. There were 180,552 proteins to be annotated, and 31,286 of which had associated blast hits and GO terms. During the blast step, it was realized that 1294 proteins had bacterial hits. These sequences were removed from downstream analyses. At the end, the obtained filtered assembly was composed by 288,380 transcripts, grouped into 216,911 genes. The mean GC content was 65.31%. The average and the median contig lengths were 778.28 bp and 803 bp, respectively. The N50 was 1204 bp. The final dataset was then translated into proteins (minimum length 50aa) to obtain a total of 179,258 protein sequences. Among these, 26,230 (14.63%) were complete (with a methionine and a stop codon), 12,559 (7.00%) started with a methionine but lacked a stop codon, 71,911 (40.11%) only had a stop codon, and 68,558 (38.24%) did not start with a methionine and did not have a stop codon. *Alexandrium tamutum* transcriptome assembly statistics are reported in Table 1.

### 2.3. Differential Expression Analysis

Differential expression analysis identified 415 transcripts with significant expression variations (|LogRealFC| > 4; *p*-value adjusted ≤0.01) in P-starvation conditions relative to control (i.e., *A. tamutum* cultured in complete K medium). Among the 415 differentially expressed genes (DEGs; of which 231 were up-regulated and 184 down-regulated) 266 transcripts had no NCBI NR assignment (of which 151 were up-regulated and 115 were down-regulated), while the remaining 149 transcripts included 80 up-regulated and 69 down-regulated genes. Functional classification analysis showed that the top GO represented classes among DEGs were ribosomes and membranes as cellular components, oxidation-reduction processes as biological process, and structural constituents of ribosomes, hydrolase activity and binding as molecular function (Figure S1). The full list of DEGs and their log2-fold changes, adjusted *P* values (padj), and GO annotations are reported in Table S1.

**Figure 1:**
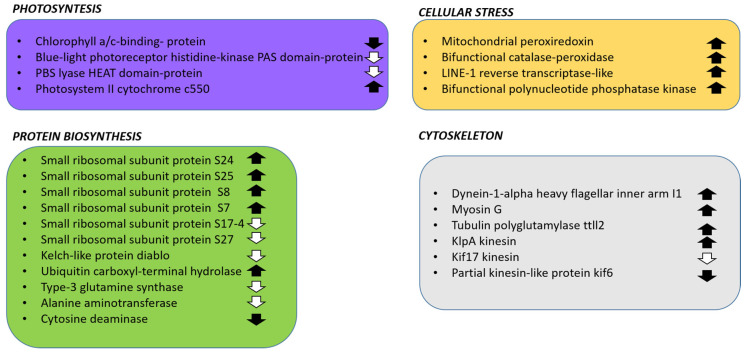
Summary of the main results for differentially expressed genes (DEGs) in the dinoflagellate *Alexandrium tamutum* cultured in phosphate starvation conditions. Up-regulated transcripts were found related to photosynthesis, cellular stress, protein biosynthesis and cytoskeleton organization biological processes; down-regulated transcripts were found related to photosynthesis, protein biosynthesis and cytoskeleton organization biological processes.

The most up-regulated annotated DEGs were the f-box/wd-40 domain-containing protein cdc4 (padj = 9.55 × 10^–5^), involved in the recognition and ubiquitination of target proteins [36]); an inosine-5-monophosphate dehydrogenase (padj = 8.79 × 10^–5^), involved in the guanine biosynthetic pathway [37]; a partial interferon-induced guanylate-binding protein 1-like (padj = 2.08 × 10^–4^), which is a large GTPase with capability of GMP production [38]. The most down-regulated annotated DEGs were a chloroplast light harvesting complex protein (padj = 3.09 × 10^–3^); the arginine n-methyltransferase 7 isoform 2 (padj = 4.43 × 10^–2^), involved in genetic imprinting [39]; a partial heat repeat-containing pbs protein (padj = 1.68 × 10^–2^), associated with phycobilisome complexes [40]. Major functional groups differentially expressed are related to photosynthesis, protein synthesis, cellular stress responses and cytoskeleton structure and functioning. Figure 1 summarizes these results, while details are reported in the following paragraphs.

#### 2.3.1. DEGs Involved in Photosynthesis

A number of transcripts involved in photosynthetic activity were differentially expressed in P-starvation: a transcript associated to a chlorophyll a/c-binding protein, a PAS domain protein constituent of the blue-light photoreceptor and a PBS lyase HEAT domain-containing protein were significantly down-regulated (-18.24-, -4.12- and -4.31-fold, respectively), while a transcript of the photosystem II cytochrome c550 was up-regulated (8.12 fold). Chlorophyll a/c-binding protein constitutes a key component of the light-harvesting complex (LHC) [41]. PAS-domain proteins act as cytosolic sensors to overall energy level of the cell [42], and PBS lyases participate to the construction of the light-harvesting phycobilisomes (PBS) [43]. The down-regulation of photosynthesis-related transcripts in P-starvation was also observed in the model diatom *Phaeodactylum tricornutum* [44], which is in general coherent with the lowered photosynthetic efficiency that was observed in green algae [45], and in higher plants [46]. On the other hand, the redox and photoprotective role of cytochrome c550 [47] can explain its up-regulation.

#### 2.3.2. DEGs Involved in Protein Synthesis

P-starvation induces a drastic proteomic reprogramming of stressed organisms as observed in the marine green alga *Micromonas commoda* [48]. In the current study, many transcripts associated with amino acid synthesis and ribosomal elements were found differentially expressed. The following transcripts associated to structural proteins belonging to the small ribosomal subunit were found up-regulated: S24 (9.54-fold), S25 (4.24-fold), S8 (7.91-fold), S7 (5.10-fold); proteins S17-4 and S27 were instead down-regulated (−4.61- and −5.43-fold respectively). Similarly, P-starvation culturing condition for the dinoflagellate *Karenia brevis* induced both over-expression and down-expression of different ribosomal proteins [49]. Specifically, the ribosomal proteins S18, L16 and S4 showed a gradual decrease in expression over the 48 h of the experiment; ribosomal proteins L2, L5 and L15 were strongly down-regulated at 48 h; ribosomal proteins S2, S5 and P0 were down-regulated over all the course of the experiment; finally, ribosomal protein S4 showed a gradual increase in expression. On the contrary, nitrogen starvation did not affect ribosomal proteins in the green alga *Botryosphaerella sudeticus* [50], while it affected the green alga *Nannochloropsis gaditana* [51]. However, various studies have also suggested extra-ribosomal functions for ribosomal proteins [52,53], but this is still unknown for microalgae. 

In addition, protein degradation was also affected by phosphate deficiency. In fact, a transcript annotated as the Kelch diablo, an adaptor protein involved in the ubiquitination pathway [54], was found down-regulated (-4.48-fold); moreover, a transcript coding a ubiquitin carboxyl-terminal hydrolase, involved in the ubiquitin monomer processing from polyubiquitin chains [55], was found up-regulated (-4.71-fold). Finally, regarding amino acids biosynthesis, two transcripts related to glutamine synthesis called type-3 glutamine synthase (-11.51- and -5.61-fold), a transcript related to alanine aminotransferase (-5.08-fold), and a transcript related to cytosine deaminase (-4.47-fold) were found down-regulated, suggesting an optimization of cellular resources, avoiding the synthesis of certain amino acids in nutrient deficiency.

#### 2.3.3. DEGs Related to Cellular Stress

The overexpression of oxidative stress-response enzymes such as superoxide dismutase (SOD), catalase (CAT) and peroxidases (POD) is often associated with the early response to P-starvation in plants [46]; on the other hand, in microalgae, the expression of these genes in stressful conditions is highly variable and there is no common trend across species [56]. In this study, P-starvation induced the overexpression of transcripts coding for a mitochondrial peroxiredoxin (10.26-fold), a bifunctional catalase-peroxidase (5.34-fold), a LINE-1 reverse transcriptase-like (4.25-fold) and a bifunctional polynucleotide phosphatase kinase (6.42-fold). Peroxiredoxin reduces hydrogen peroxide via the oxidation of a redox-active cysteine residue [57] and its expression levels were up-regulated by light-induced stress in the raphidophyte *Chattonella marina* [58] and increased in *Chlamydomonas reinhardtii* in iron starvation conditions [59]. Bifunctional catalase-peroxidase, while mainly found in bacteria [60], was also characterized in some microalgae, such as the dinoflagellate *Prorocentrum minimum* [61] and the green alga *Volvox carteri* f. *nagariensis* (GenBank accession no. XP_002956382). 

The LINE-1 reverse transcriptase is associated with LINE-1 retrotransposon propagation [62] and, while not directly involved in stress response, it could have a role in microalgal genetic adaptation to stressful environments, as observed in other eukaryotes [63]. For the same reason, the bifunctional polynucleotide phosphatase kinase could be involved in DNA repair [64], and was found to be over-expressed in *A. tamutum*.

#### 2.3.4. DEGs Related to Cytoskeleton

While phosphate deficiency is well studied in higher plants, where it causes stunting [50], there is less evidence of the effects of such stressful conditions for microalgae. In this transcriptome, P-starvation induced the up-regulation of transcripts coding for a dynein annotated as (“dynein-1-alpha heavy flagellar inner arm I1”), a myosin G, a tubulin polyglutamylase ttll2 and a klpA kinesin (4.47-, 4.84-, 4.29-, 8.16-fold, respectively). Dyneins are microtubular motor proteins and contain a variable number of heavy chains [65]; furthermore, in the dinoflagellate *Prorocentrum donghaiense*, 16 dynein heavy chain (DHC) genes were substantially up-regulated [66] in P-starvation, suggesting a promotion of organelle traffic and cellular motility. Myosin G transcripts codes for a motor domain of the actin-based microtubular protein myosin [67]; tubulin polyglutamylase ttll2 is involved in post-transcriptional tubulin polyglutamylation, which regulates the electrostatic interaction between microtubules and MAPs [68]); finally, kplA is a bidirectional microtubule motor protein [69]. P-starvation also induced the down-regulation of transcripts coding a kif17 kinesin (-13.53-fold) and a kif6 kinesin (-4.05-fold); kinesin kif17 is a motor protein involved in intraflagellar transport [70], while the kif6 family is involved in intracellular organelle transport [71]. These transcriptional variations suggest a plasticity of the *A. tamutum* cytoskeleton. 

### 2.4. Sequences Coding Enzymes Involved in Toxin Synthesis

In order to search in the transcriptome of *A. tamutum* for sequences related to toxin synthesis, the transcripts related to the saxitoxins biosynthetic pathway found by Hackett and colleagues in *A. tamarense* CCMP1598 (Group IV) ([19] deposited in GenBank with the accession numbers JV310009-JV310320) were used as queries. Of the 258 contigs found by Hackett et al. (related to the cyanobacteria genes sxtA, sxtB, sxtD, sxtF/M, sxtG, sxtH/T, sxtI, sxtL, sxtN, sxtS, sxtU and sxtX), only 18 sequences related to sxtA, sxtG and sxtU genes were found in the current *A. tamutum* transcriptome. SxtA is the starting gene of SXT-synthesis in cyanobacteria and has a polyketide synthase (PKS)-like structure. It is characterized by four catalytic domains: S-adenosyl-methionine- (SAM) dependent methyltransferase (sxtA1), which adds a methyl group to acetyl ACP forming propionyl ACP, GCN5-related N-acetyltransferase (sxtA2), acyl carrier protein (sxtA3), and a class II aminotransferase (sxtA4) [18,72]. The sxtA4 domain catalyses a Claisen condensation between arginine and propionyl-ACP, producing the first ‘A’ intermediate [4,72]. Following the reactions catalysed by sxtA, sxtG catalyses the transfer of a guanidine group from a second arginine to the growing SXT backbone forming a ‘B intermediate’. Finally, sxtU encodes for a short-chain alcohol dehydrogenase. This enzyme is thought to reduce the terminal aldehyde group on C1 of the STX precursor (tricyclic alcohol intermediate) in the SXT biosynthetic pathway that was theorized in cyanobacteria and dinoflagellates [73,74]. The 18 matching sequences in the transcriptome are listed in Table 2. Each transcript was further analyzed by using the NCBI Conserved Domain (CDD) database, confirming the identity of the encoded domains found in *A. tamarense* CCMP1598. In addition, none of the identified sequences showed differential expression rates under P-starvation. 

**Figure 2:**
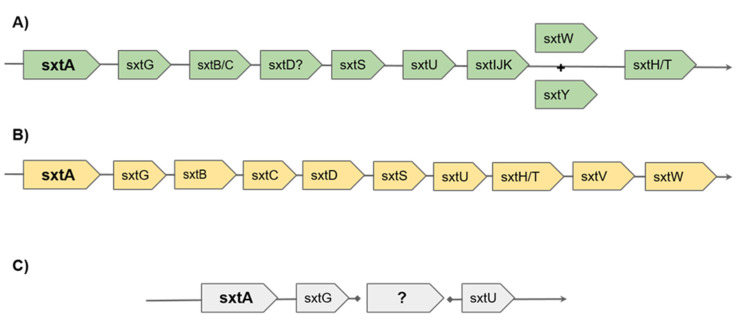
Sxt biosynthetic pathway: (**A**) sxt biosynthetic pathway as theorized by Kellmann in the cyanobacteria *Cylindrospermopsis raciborskii T3* [72]; (**B**) biosynthetic pathway as theorized by Zhang et al. [75] and reviewed by Akbar et al. in dinoflagellates [4]; (**C**) sxt incomplete pathway as observed from the transcripts present in the current *A. tamutum* transcriptome.

In dinoflagellates, the biosynthesis of SXT involves the genes sxtA, sxtG, sxtB, sxtD, sxtS, sxtU, sxtH/T and sxtI (as recently reviewed by Akbar et al; Figure 2). The absence of transcripts homologous to sxtB, sxtD, sxtS, sxtH/T and sxtI in this transcriptome could explain the absence of SXT detection in the studied clone [6]. However, such data cannot exclude the synthesis of a toxic intermediate. Regarding the expression levels of sxtA, sxtG and sxtU, these genes did not change at different growth stages in *A. catenella* [75], but were down-regulated in nitrogen and phosphate deficiency in *A. minutum* [76]. 

However, *Alexandrium* spp. are known to harbour intracellular amounts of phosphate [5,77], and many authors observed an increased toxicity and toxin production rate in P-starvation: for example, Han et al. [78] found that P-starvation increased the toxicity of *A. pacificum* up to 20 times in comparison to nitrogen starvation cultures. In the literature, various studies reported that nutrient starvation induced an increase in toxicity of other microalgae, but such effects vary among species: toxicity increased in nitrogen starvation (N-starvation) in the dinoflagellate *Karenia brevis* [79], whereas it decreased in the dinoflagellate *Protogonyaulax tamarensis* and increased dramatically in P-starvation [80]. Similar results have also been observed for other microalgae, such as the diatom *Pseudo-nitzschia* sp. with increased toxicity when cultured under starvation of different micro- and macro-nutrients [81].

### 2.5. Structure Prediction of Proteins Encoded by sxt Genes

**Figure 3:**
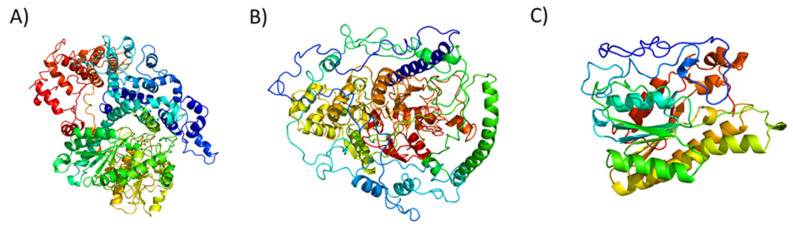
PDB sxt protein models: (**A**) sxtA N-Terminus; (**B**) sxtG Amidinotransferase; (**C**) sxtU Short-chain alcohol dehydrogenase. Individual PDB models are available in Files S1, S2 and S3.

In order to further investigate sxt genes at the protein structure levels, in silico prediction of their three-dimensional structure was performed using the fold recognition approach: the in silico modelling of the three-dimensional structures of the sxt gene products found in the *A. tamutum* transcriptome were performed by using the PHYRE2 program [82]. The modelled structures had PHYRE2 confidence scores of 100%. The transcript homologous for sxtA C-terminus possessed an incomplete amino acid sequence, and was excluded from further analyses. The results of the in silico modelling are reported in Table 3 and in Figure 3. 

Although the genes involved in *sxt* biosynthesis have been found in many cyanobacteria and dinoflagellates, there are few three-dimensional protein models. The crystallographic structure of an amidinotransferase annotated as sxtG in the cyanobacterium *Microseira wollei* has been recently published [83], highly different in amino acid number and sequence from the sxtG found in the present work. To our knowledge, these are the first in silico predictions of three-dimensional structures of sxt gene products in dinoflagellates.

### 2.6. Other Toxin-Related Transcripts 

Other genes putatively related to toxin compound production were annotated in the *A. tamutum* transcriptome (Table 4). Two transcripts related to aflatoxin metabolism (one annotated as “aflatoxin b1 aldehyde reductase member 4-like”, the other as “aflatoxin b1 aldehyde reductase member 2”), one as “gliotoxin biosynthesis protein, two annotated as “toxicos en levadura”, and three transcripts simply annotated as “toxin biosynthesis protein” were found. These sequences were manually checked on NCBI databases in order to unravel their potential role.

Both aflatoxin-related transcripts showed a conserved domain associated to Aldo-keto reductases that reduce aldehydes and ketones to primary and secondary alcohols [84]. Such enzymes are produced by many organisms, with a broad range of substrates and functions (biosynthesis of hormones and small metabolites, detoxification mechanisms), and were suggested as potential chemopreventive agents [85]. Another aldehyde reductase has also been reported in the green algae *Nannochloropsis gaditana* [86], but to our knowledge this is the first case of an aldehyde reductase of this family in dinoflagellates. 

GGCT (gamma-glutamyl cyclotransferase)-like domains in the transcript annotated as “gliotoxin biosynthesis protein” that are involved in the metabolism of different molecules from gamma-glutamyl dipeptides, and in glutathione homeostasis [87] were also found. Such domains have also been reported in the green algae *Coccomyxa subellipsoidea* [88], but never in a dinoflagellate.

The two “toxicos en levadura” transcripts (that shared a 96% identity) possessed a conserved domain related to the HRD ubiquitin ligase complex (involved in quality control ubiquitination and protein turnover [89], expressed in *Arabidopsis thaliana* after exposure to chitin or inactivated crude cellulase preparations, probably as part of its triggered immunity (original Gene ID: 820924).

Finally, the three sequences annotated as “toxin biosynthesis protein” possess a domain related to the 2-Oxoglutarate-Fe(II) oxygenase superfamily. In plants, these enzymes are known to be the most versatile in nature, catalysing a large diversity of biologically relevant reactions, including the biosynthesis of toxins and other metabolites [90,91]. Prolyl 4-hydroxylases (P4Hs, catalyses the hydroxylation of peptidyl prolines) belong to this superfamily that was previously characterized in *Chlamydomonas reinhardtii* [92], but the variety of substrates of these enzymes in microalgae needs further exploration. None of these transcripts showed differential expression under P-starvation. The analyzed sequences, their CDD outputs and potential functions are shown in Table 4.

Moreover, other sequences annotating polyketide synthases (PKS) or nonribosomal peptide synthases (NRPS) were searched in the transcriptome. Both PKS and NRPS produce secondary metabolites (polyketides and peptides, respectively) via their modular enzymatic assembly lines. Their products have already been suggested to be related to antibacterial, antifungal, antipredator, allelopathic and anticancer activities [21,93,94,95]. In the studied transcriptome, a total of 44 transcripts were annotated as “polyketide synthase”, and 5 annotated as “nonribosomal peptide synthetase polyketide synthase hybrid” were found. None of these showed differential expression under P-starvation.

All PKS share different essential domains: acyltransferase domain (AT), β-ketosynthase domain (KS), β-ketoacyl reductase (KR), enoyl reductase (ER), methyl transferases (MT), thioesterases (TE), dehydrogenase (DH) and acyl carrier protein (ACP) domains. NRPS are composed by a variable number of modules, containing an adenylation (A), a peptide acyl-carrier (T), and a condensation (C) domain, plus different tailoring domains [96]. Finally, PKS-NRPS hybrid genes produce peptide–polyketide metabolites, in which polyketides are fused to an amino acid by an amide bond [97]. 

According to our phylogenetic analysis, most of the transcripts annotated as polyketide synthases were type I PKS (41 out of 44) and clustered together with other dinoflagellate PKS sequences in several highly supported clades (red dots, Figure S2). Despite the fact that type I PKSs are typically multifunctional enzymes organized into modules, each harbouring a set of distinct domains, we only found single domain type I PKSs, as reported for other toxic dinoflagellate studies [98,99,100]. The three remnant PKS transcripts were found in the same clade containing type III PKS sequences (chalcone and stilbene synthases, Figure S2), recently reported in other dinoflagellate transcriptomes [101]. This study constitutes the first report for type III PKSs in *A. tamutum*. The full list of PKS found in *A. tamutum* transcriptome is reported in Table S2.

A CDD search of PKS/NRPS hybrid-annotated transcripts evidenced two “Acyl-CoA synthetase (AMP-forming)/AMP-acid ligase II”, two “adenylation domain of nonribosomal peptide synthetases (NRPS)” and one “non-ribosomal peptide synthetase component F + Phosphopantetheine attachment site”. PKS genes have been identified in several microalgae (e.g., *Azadinium spinosum, Gambierdiscus* spp., *Amphidinium carterae, Tetraselmis suecica* [95,102,103,104]), while metabolites theorized to derive from hybrid NRPS/PKS gene clusters have been reported in *Karenia brevis* [105]. The wide range of suggested enzymatic reactions and possible natural products that may be produced, make these microalgal species interesting targets for future studies with biotechnological applications [106].

## 3. Materials and Methods

### 3.1. Cell Culturing and Harvesting

*Alexandrium tamutum* (strain FE107 from the Stazione Zoologica culture collection) was cultured in Keller medium (K) [107]. Experimental culturing for both control and P-starvation conditions was performed in 2 litre polycarbonate bottles (each condition was performed in biological triplicates), constantly bubbled with air filtered through 0.2 µm membrane filters. For the control condition, normal K medium was used, while for the P-starvation experiment, the K medium was prepared with low concentrations of phosphate (0.5 μM PO_4_^2−^ rather than 36 μM PO_4_^2−^ of the control condition). Cultures were kept in a climate chamber at 19 °C on a 12:12 h light:dark cycle at 100 µmol photons m^−2^ s^−1^. Initial cell concentration for each bottle was about 5000 cells/mL. Culture growth was monitored daily by sampling 2 mL of culture, and fixing with one drop of Lugol (final concentration of about 2%, *v*/*v*) and counting cell numbers in a Bürker counting chamber under an Axioskop 2 microscope (20×) (Carl Zeiss GmbH, Oberkochen, Germany) (as in Elagoz et al. [108]). The growth curve of *Alexandrium tamutum* (in control and P-starvation conditions) is available in Figure S3. Culture aliquots (50 mL) were sampled during the stationary phase on the fifth growth day (on the same day and at the same time of day for each replicate and for each condition, in order to avoid possible interference due to circadian rhythms), and centrifuged for 15 min at 4 °C at 1900× *g* (Eppendorf, 5810R, Hamburg, Germany). For RNA extractions, pellets (triplicates for each condition) were re-suspended in 500 µL of TRIZOL© (Invitrogen, Carlsbad, CA), incubated for 2–3 min at 60 °C until completely dissolved, and kept at −80°C. 

### 3.2. RNA Extraction

For RNA extraction, *A. tamutum* cells, previously frozen in TRIZOL®, were lysed using half a spatula of glass beads (about 200 mg; Sigma-Aldrich, Milan, Italy) for each 2 mL tube, incubating and mixing tubes for 10 min at 60 °C, and then at maximum speed in the Thermo Shaker BS100 (Biosan, Rīga, Latvia). The RNA was then extracted following TRIZOL® manufacturer’s instructions. RNA quantity and purity were assessed by Nano-Drop (ND-1000 UV-Vis spectrophotometer; NanoDrop Technologies, Thermo Fisher Scientific, Waltham, MA, USA) measuring the absorbance at 260 nm, and the 260/280 nm and 260/230 nm ratios (both ratios were about 2.0). RNA quality was further evaluated by gel electrophoresis that showed intact RNA, with sharp ribosomal bands. Finally, total RNA quality was evaluated by measuring the RNA integrity number (RIN) with Agilent 2100 Bioanalyzer (Agilent Technologies, Inc., Santa Clara, CA, USA). High quality (RIN > 8) RNA was used for RNAseq for both control and P-starvation conditions.

### 3.3. Library Preparation and Sequencing

RNA sequencing, including sample quality control, was performed by Genomix4life S.R.L. (Baronissi, Salerno, Italy). Indexed libraries were prepared from 2 ug/ea purified RNA with TruSeq Stranded mRNA Sample Prep Kit (Illumina, CA, USA), according to the manufacturer’s instructions. Libraries were quantified using the Agilent 2100 Bioanalyzer (Agilent Technologies) and were then pooled so that each index-tagged sample was present in equimolar amounts (final concentration of the pooled samples was 2nM). The pooled samples were subjected to cluster generation and sequencing using an Illumina HiSeq 2500 System (Illumina, CA, USA) in a 2 × 100 paired-end format at a final concentration of 8 pmol. The raw sequence files generated (fastq files) underwent quality control analysis using FastQC [109].

### 3.4. Transcriptome Assembly and Annotation

Illumina paired-end 100 bp reads were processed to produce the transcriptome assembly. Reads are freely available under the series entry PRJNA632001 in the Sequence Read Archive (SRA) NCBI database [110]. Raw reads were trimmed and clipped with BBDuk [111] setting a minimum Phred-like quality of 25 and a minimum length of 35 nucleotides. The quality of the reads before and after trimming was checked with the software FASTQC [109]. High quality reads were then normalized with Trinity [112] using the options: -SS_lib_type RF -pairs_together -max_cov 50. De novo transcriptome assembly was then performed with Trinity using the options: -SS_lib_type RF -no_normalize_reads -min_kmer_cov 1 -KMER_SIZE 32. Transcriptome redundancy was removed with CD-HIT-EST [113] using the following options: -r 0 -g 1. A filter for contaminants was performed by BLASTing the transcripts against the NCBI nr database, discarding all the sequences having a significant hit (*e* value ≤ 0.0001) against bacteria or metazoa. The completeness of the assembly was checked against the core eukaryotic genes database [34]. The distribution of the GC content was evaluated with a *z*-test, performed with the z.test function in R. In silico translation was performed with TransDecoder [114] and the presence of repetitive elements was analysed with Repeat Masker (Statistics in Table S3), while functional annotation was performed with Blast2GO software [115].

### 3.5. Transcriptome Expression Quantification and Differential Expression Analysis

Transcript expression quantification was performed using Express (v 1.5.1) [116] after mapping the reads against the assembly with STAR [117]. Posterior counts were used as input to perform transcript differential expression analysis with EBSeq [118]. Transcripts with expression variations with |LogRealFC| > 4 and *p* value adjusted ≤0.01 were considered significant.

### 3.6. In Silico Protein Modelling

The NCBI CDD database search interface (https://www.ncbi.nlm.nih.gov/Structure/cdd/wrpsb.cgi) was first used to identify the conserved protein domains of the amino acid sequences. PHYRE2 [82] was used for the three-dimensional (3D) in silico protein modelling of the *sxt* genes: sxtA N-terminus, sxtG and sxtU.

### 3.7. Phylogenetic Tree

For the transcripts (44) that were annotated as polyketide synthases by B2GO analysis, a phylogenetic tree was inferred to assess their evolutionary relatedness. We included in our analysis type I and type III PKS sequences of other dinoflagellates from the literature (Table S4). Transcripts were aligned using COBALT [119] and poorly aligned regions were removed with trimAl v1.2 [120] using the automated1 option. The final alignment included 64 sequences and 815 aa (File S4). A maximum likelihood phylogenetic tree was then inferred in PhyML [121] using the evolution model (LG+G+F) suggested by Smart Model Selection (SMS) [122]. Support to nodes was calculated using the Shimodaira–Hasegawa-like (aLRT SH-like) procedure [123]. The resulting tree was visualised and graphically edited in FigTree v1.4.3 [124].

## 4. Conclusions

Dinoflagellates are known to produce various toxins and to give rise to harmful algal blooms, with important consequences for human health and economies. The increase in the frequency and intensity of harmful algal blooms and the toxins these produce has been related to global warming [3]. Hence, there is an increased need to study toxin biosynthetic pathways and the enzymes involved in their metabolism, in order to rapidly detect harmful algal blooms, monitor their distribution and toxicity, contrast these occurrences, and when possible, reduce their detrimental effects.

In this study, we focused on a clone of the dinoflagellate *A. tamutum* which has previously shown reproductive impairment on crustacean copepods [6] and antiproliferative activities on human cancer cell lines [17], but which did not produce saxitoxins. We nonetheless performed an in silico search of enzymes that can be involved in toxin synthesis (i.e., saxitoxin synthesis-related genes) or other enzymes that can be involved in the synthesis of potentially toxic compounds (e.g., polyketide synthases). Results showed the presence of transcripts related to only three genes of *sxt* synthesis (i.e., sxtA sxtG and sxtU), but also the presence of other enzymes that can be involved in toxin production (e.g., aldo-keto reductases, 2-Oxoglutarate-Fe(II) oxygenases, PKS and PKS/NRPS-related transcripts), with or without exposure to stressful nutrient starvation. 

These data suggest the presence of possible toxic compounds, other than saxitoxins, which may be responsible for the toxic and antiproliferative activity of *A. tamutum*, as reported for other *Alexandrium* spp. which produced “uncommon” toxins [28]. Such findings indicate that the chemistry of toxin production in dinoflagellates is very complex and requires in depth new studies in both the ecological and drug discovery fields in order to identify novel chemical mediators in the marine environment and new lead compounds that can be developed as pharmaceuticals. Microalgae have already shown to have several bioactivities for the treatment of human pathologies, such as anticancer, anti-inflammatory, anti-diabetes, antioxidant, anti-tuberculosis, anti-epilepsy, anti-hypertensive, anti-atherosclerosis and anti-osteoporosis activities [95,125,126,127,128]. In particular, *Alexandrium minutum* has been shown to be active on human lung cancer cells [129], *Alexandrium andersoni* induces cell death in lung and colorectal tumour cell lines [130], while *Alexandrium tamutum* was active on human melanoma cell lines [17]. Altogether, these data suggest that *Alexandrium* spp. may produce metabolites that can have anticancer applications and are worthy of further investigation. 

## Figures and Tables

**Table 1:** *Alexandrium tamutum* transcriptome assembly statistics.

Number of Genes	216,911
Number of transcripts	288,380
Percent GC content	65.31
Contig N50	1204
Median contig length	474
Average contig length	778.28
Number of proteins	179,258
Number of complete proteins	26,230
Number of partial proteins	153,028

**Table 2:** Sxt genes found in *A. tamutum* transcriptome. For each gene the encoded protein, the relative *A. tamarense* query from [19], the transcript ID and the best matching NCBI BLAST results are listed.

Gene	Encoded Protein	Original Sequence Code in Hackett, 2013	Sequence ID and Internal BLAST Results	NCBI BLAST Best Match and Accession Number
sxtA	Phosphopantetheine attachment site (ACP in PKS)	sxtA, N terminus Contig93306	>TR16074|c0_g1_i1 Length = 3230 Score = 402 bits (203), Expect = e-110 Identities = 443/523 (84%) Strand = Plus/Minus	*Alexandrium catenella* strain CS319 sxtA-like (sxtA) gene, partial sequence; KM100452.1
sxtA	Aspartate aminotransferase	sxtA, C terminus Aspartate aminotransferase Contig106704	>TR2168|c0_g2_i2 Length = 1724 Score = 371 bits (187), Expect = e-101 Identities = 352/407 (86%) Strand = Plus/Plus	*Aminobacter* sp. MSH1 chromosome, complete genome; CP026265.1
sxtG	Amidinotransferase	sxtG, Amidinotransferase Contig22175	>TR24523|c0_g4_i1 Length = 3296 Score = 1292 bits (652), Expect = 0.0 Identities = 1483/1760 (84%) Strand = Plus/Plus	*Ensifer adhaerens* strain Casida A plasmid pCasidaAA, complete sequence; CP015881.1
sxtU	Short-chain alcohol dehydrogenase	sxtU, Short-chain alcohol dehydrogenase Contig1416	>TR101104|c3_g5_i1 Length = 1221 Score = 712 bits (359), Expect = 0.0 Identities = 704/819 (85%) Strand = Plus/Minus	*Polyangium brachysporum* strain DSM 7029, complete genome; CP011371.1
sxtU	Short-chain alcohol dehydrogenase	sxtU, Short-chain alcohol dehydrogenase Contig22159	>TR119378|c0_g1_i1 Length = 1982 Score = 696 bits (351), Expect = 0.0 Identities = 648/747 (86%) Strand = Plus/Minus	*Sandaracinus amylolyticus* strain DSM 53668, complete genome; CP011125.1
sxtU	Short-chain alcohol dehydrogenase	sxtU, Short-chain alcohol dehydrogenase Contig22852	>TR140478|c0_g2_i1 Length = 1469 Score = 381 bits (192), Expect = e-104 Identities = 381/444 (85%) Strand = Plus/Minus	*Emiliania huxleyi* CCMP1516 hypothetical protein mRNA; XM_005787014.1
sxtU	Short-chain alcohol dehydrogenase	sxtU, Short-chain alcohol dehydrogenase Contig22852	>TR140478|c0_g1_i1 Length = 1457 Score = 381 bits (192), Expect = e-104 Identities = 381/444 (85%) Strand = Plus/Minus	*Emiliania huxleyi* CCMP1516 hypothetical protein mRNA; XM_005787014.1
sxtU	Short-chain alcohol dehydrogenase	sxtU, Short-chain alcohol dehydrogenase Contig24779	>TR3419|c0_g1_i1 Length = 1019 Score = 559 bits (282), Expect = e-158 Identities = 585/686 (85%) Strand = Plus/Minus	*Emiliania huxleyi* CCMP1516 hypothetical protein partial mRNA; XM_005768652.1
sxtU	Short-chain alcohol dehydrogenase	sxtU, Short-chain alcohol dehydrogenase Contig31067	>TR32351|c0_g1_i1 Length = 691 Score = 394 bits (199), Expect = e-108 Identities = 415/487 (85%) Strand = Plus/Minus	*Stigmatella aurantiaca* DW4/3-1, complete genome; CP002271.1
sxtU	Short-chain alcohol dehydrogenase	sxtU, Short-chain alcohol dehydrogenase Contig34277	>TR63446|c0_g4_i1 Length = 1023 Score = 533 bits (269), Expect = e-150 Identities = 551/645 (85%) Strand = Plus/Plus	*Caulobacter mirabilis* strain FWC 38 chromosome, complete genome; CP024201.1
sxtU	Short-chain alcohol dehydrogenase	sxtU, Short-chain alcohol dehydrogenase Contig34756	>TR70482|c0_g1_i1 Length = 1181 Score = 486 bits (245), Expect = e-136 Identities = 587/701 (83%) Strand = Plus/Minus	*Anopheles gambiae* str. PEST AGAP008667-RA (AgaP_AGAP008667), partial mRNA; XM_314766.4
sxtU	Short-chain alcohol dehydrogenase	sxtU, Short-chain alcohol dehydrogenase Contig44170	>TR142607|c0_g1_i1 Length = 1418 Score = 593 bits (299), Expect = e-168 Identities = 638/751 (84%) Strand = Plus/Minus	*Chromobacterium vaccinii* strain XC0014 chromosome, complete genome; CP022344.1
sxtU	Short-chain alcohol dehydrogenase	sxtU, Short-chain alcohol dehydrogenase Contig44865	>TR84807|c0_g1_i1 Length = 1081 Score = 422 bits (213), Expect = e-117 Identities = 450/529 (85%) Strand = Plus/Minus	*Phenylobacterium zucineum* HLK1, complete genome; CP000747.1
sxtU	Short-chain alcohol dehydrogenase	sxtU, Short-chain alcohol dehydrogenase Contig64321	>TR80533|c0_g3_i1 Length = 989 Score = 472 bits (238), Expect = e-131 Identities = 490/574 (85%) Strand = Plus/Plus	*Bradyrhizobium diazoefficiens* DNA, complete genome, strain: NK6; AP014685.1
sxtU	Short-chain alcohol dehydrogenase	sxtU, Short-chain alcohol dehydrogenase Contig64321	>TR80533|c0_g2_i1 Length = 916 Score = 472 bits (238), Expect = e-131 Identities = 490/574 (85%) Strand = Plus/Plus	*Bradyrhizobium diazoefficiens* DNA, complete genome, strain: NK6; AP014685.1
sxtU	Short-chain alcohol dehydrogenase	sxtU, Short-chain alcohol dehydrogenase Contig86383	>TR142098|c0_g1_i2 Length = 1193 Score = 448 bits (226), Expect = e-124 Identities = 427/494 (86%) Strand = Plus/Minus	PREDICTED: *Aegilops tauschii subsp. tauschii momilactone A* synthase-like (LOC109773470), mRNA; XM_020332162.1
sxtU	Short-chain alcohol dehydrogenase	sxtU, Short-chain alcohol dehydrogenase Contig86383	>TR142098|c0_g1_i3 Length = 936 Score = 424 bits (214), Expect = e-117 Identities = 424/494 (85%) Strand = Plus/Minus	PREDICTED: *Aegilops tauschii subsp. tauschii momilactone A* synthase-like (LOC109773470), mRNA; XM_020332162.1
sxtU	Short-chain alcohol dehydrogenase	sxtU, Short-chain alcohol dehydrogenase Contig97277	>TR33997|c0_g1_i3 Length = 1012 Score = 555 bits (280), Expect = e-157 Identities = 616/728 (84%) Strand = Plus/Plus	*Stenotrophomonas maltophilia* strain AA1, complete genome; CP018756.1

**Table 3:** Report of PHYRE2 modelling. The *A. tamutum* sxt genes (and transcript code), template (protein of known structure used for the in silico modelling), its protein data bank (PDB) code, confidence (probability that the sequence and template are homologous), % id (% of identity) and NCBI Conserved Domain (CDD) search output are reported.

*A. tamutum sxt* Putative Protein	Template (PDB Code)	Confidence	% id	CDD Search Output
sxtA N-Terminus; TR16074|c0_g1_i1	Methyltransferase (6B3A)	100%	24%	Phosphopantetheine attachment site
sxtG Amidinotransferase; TR24523|c0_g4_i1	Arginine deaminase (1RXX)	100%	32%	Aminotransferase superfamily; Arginine deiminase
sxtU Short-chain alcohol dehydrogenase; TR101104|c3_g5_i1	Glucose dehydrogenase (1GEE)	100%	46%	Rossmann-fold NAD(P)(+)-binding proteins

**Table 4:** Potential toxin-related genes found in the *A. tamutum* transcriptome. For each gene, the automatic transcript annotation, the transcript ID, the output of CDD search and their potential function are listed.

Transcript Automatic Annotation	Transcript ID	CDD Search Output	Potential Function
aflatoxin b1 aldehyde reductase member 2	TR29768|c0_g1_i1	Aldo-keto reductase (AKR)	Metabolites detoxification; carbon metabolism
aflatoxin b1 aldehyde reductase member 4-like	TR93512|c0_g1_i1	Aldo-keto reductase (AKR)	Metabolites detoxification; carbon metabolism
gliotoxin biosynthesis protein	TR57386|c0_g1_i1	GGCT-like domain	Metabolites biosynthesis; glutathione homeostasis
“toxicos en levadura”	TR119771|c0_g1_i1	HRD ubiquitin ligase complex	Ubiquitination; triggered immunity
“toxicos en levadura”	TR119771|c0_g2_i1	HRD ubiquitin ligase complex	Ubiquitination; triggered immunity
toxin biosynthesis protein	TR47414|c1_g1_i2	2OG-Fe(II) oxygenase superfamily	Toxins/Metabolites production
toxin biosynthesis protein	TR120505|c0_g1_i1	2OG-Fe(II) oxygenase superfamily	Toxins/Metabolites production
toxin biosynthesis protein	TR120505|c0_g1_i2	2OG-Fe(II) oxygenase superfamily	Toxins/Metabolites production

## Supplementary Materials

The following are available online at https://www.mdpi.com/1660-3397/18/8/386/s1, Table S1: Differential Expressed Genes; Table S2: PKS genes; Table S3: RepeatMasker statistics results; Table S4: List of dinoflagellate PKS sequences from the literature utilized for phylogenetic inference. Figure S1: Histograms of GO classifications showing sequence distribution of the annotated differentially expressed genes within the cellular component (A), biological process (B) and molecular function (C). The y-axis indicates the number of sequences for each category; Figure S2: Maximum likelihood phylogenetic tree of transcripts identified as polyketide synthases by Blast 2GO analysis. Support to nodes (coloured circles) was inferred using the Shimodaira–Hasegawa-like test; Figure S3: *A. tamutum* growth curve; File S1: PDB file of *sxtA*; File S2: PDB file of *sxtG*; File S3: PDB file of *sxtU*; File S4: Alignment of PKS amino acid sequences.
