# Supplementary material for: De novo Transcriptome of the Non-saxitoxin Producing Alexandrium tamutum Reveals New Insights on Harmful Dinoflagellates
Source: Mar Drugs. 2020 Jul 24;18(8):386. doi: 10.3390/md18080386 (PMC7460133; doi:10.3390/md18080386)
Supplement: Supplementary file 1 [file marinedrugs-18-00386-s001.zip › Supplementary files/Supplementary Table 4.docx]

**Supplementary Table 4. List of dinoflagellate PKS sequences from literature utilized for phylogenetic inference.**

| **Accession number/**  **Transcript code** | **Annotation** | **Species** | **Reference** |
| --- | --- | --- | --- |
| AFW98413.1 | Type I PKS | *Alexandrium ostenfeldii* | Eichholz et al. (2012) |
| AFW98411.1 | Type I PKS | *Alexandrium ostenfeldii* | Eichholz et al. (2012) |
| AFW98412.1 | Type I PKS | *Alexandrium ostenfeldii* | Eichholz et al. (2012) |
| AIW63288.1 | PKS | *Azadinium spinosum* | Meyer et al. (2015) |
| AQS99211.1 | Type I PKS | *Gambierdiscus excentricus* | Kohli et al. (2017) |
| AQS99217.1 | Type I PKS | *Gambierdiscus excentricus* | Kohli et al. (2017) |
| AQS99218.1 | Type I PKS | *Gambierdiscus excentricus* | Kohli et al. (2017) |
| AQS99241.1 | Type I PKS | *Gambierdiscus excentricus* | Kohli et al. (2017) |
| AQS99271.1 | Type I PKS | *Gambierdiscus excentricus* | Kohli et al. (2017) |
| AQS99289.1 | Type I PKS | *Gambierdiscus excentricus* | Kohli et al. (2017) |
| AQS99293.1 | Type I PKS | *Gambierdiscus excentricus* | Kohli et al. (2017) |
| AQS99216.1 | Type I PKS | *Gambierdiscus polynesiensis* | Kohli et al. (2017) |
| AQS99228.1 | Type I PKS | *Gambierdiscus polynesiensis* | Kohli et al. (2017) |
| AQS99236.1 | Type I PKS | *Gambierdiscus polynesiensis* | Kohli et al. (2017) |
| AQS99269.1 | Type I PKS | *Gambierdiscus polynesiensis* | Kohli et al. (2017) |
| AQS99314.1 | Type I PKS | *Gambierdiscus polynesiensis* | Kohli et al. (2017) |
| BAT21728.1 | PKS | *Karenia mikimotoi* | Kimura et al. (2015) |
| CAMPEP_0189923024 | Type III PKS | *Kryptoperidinium foliaceum* CCMP1326 | De Luca and Lauritano (2020) |
| CAMPEP_0199953780 | Type III PKS | *Durinskia baltica CSIRO_CS38* | De Luca and Lauritano (2020) |
| CAMPEP_0199957422 | Type III PKS | *Durinskia baltica*  *CSIRO CS38* | De Luca and Lauritano (2020) |

**References**

De Luca, D., & Lauritano, C. (2020). In Silico Identification of Type III PKS Chalcone and Stilbene Synthase Homologs in Marine Photosynthetic Organisms. *Biology*, *9*(5), 110.

Eichholz, K., Beszteri, B., & John, U. (2012). Putative monofunctional type I polyketide synthase units: a dinoflagellate-specific feature?. *PLoS One*, *7*(11), e48624.

Kimura, K., Okuda, S., Nakayama, K., Shikata, T., Takahashi, F., Yamaguchi, H., ... & Tomaru, Y. (2015). RNA sequencing revealed numerous polyketide synthase genes in the harmful dinoflagellate *Karenia mikimotoi*. *PLoS One*, *10*(11), e0142731.

Kohli, G. S., Campbell, K., John, U., Smith, K. F., Fraga, S., Rhodes, L. L., & Murray, S. A. (2017). Role of modular polyketide synthases in the production of polyether ladder compounds in Ciguatoxin‐producing *Gambierdiscus polynesiensis* and *G. excentricus* (Dinophyceae). *Journal of Eukaryotic Microbiology*, *64*(5), 691-706.

Meyer, J. M., Rödelsperger, C., Eichholz, K., Tillmann, U., Cembella, A., McGaughran, A., & John, U. (2015). Transcriptomic characterisation and genomic glimps into the toxigenic dinoflagellate *Azadinium spinosum*, with emphasis on polyketide synthase genes. *BMC genomics*, *16*(1), 27.
