# Supplementary figures and images for: De novo Transcriptome of the Non-saxitoxin Producing Alexandrium tamutum Reveals New Insights on Harmful Dinoflagellates
Source: Mar Drugs. 2020 Jul 24;18(8):386. doi: 10.3390/md18080386 (PMC7460133; doi:10.3390/md18080386)

A. Cellular components

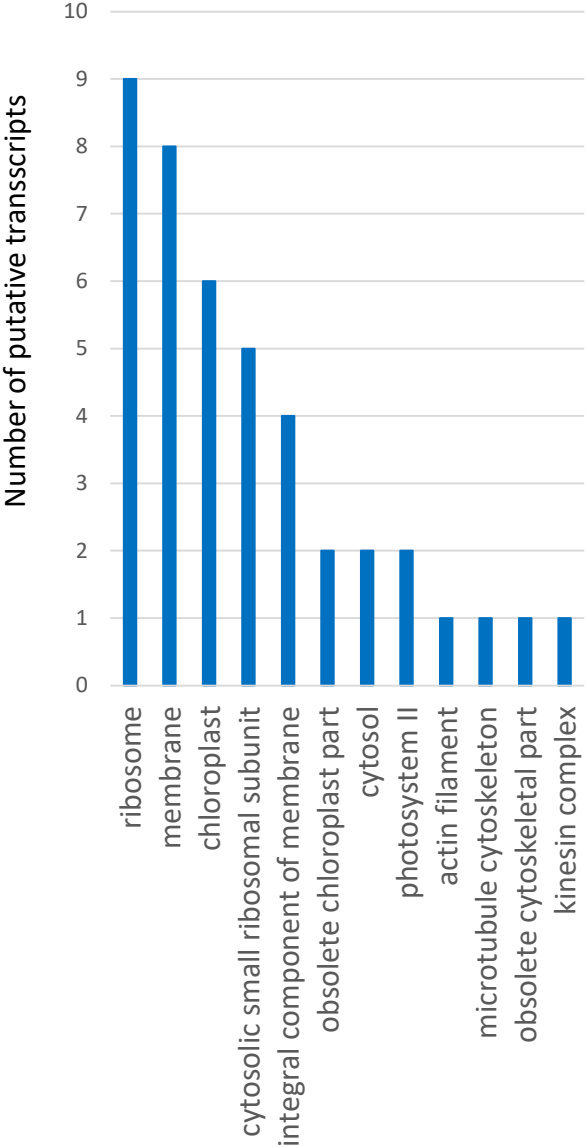

B. Biological process

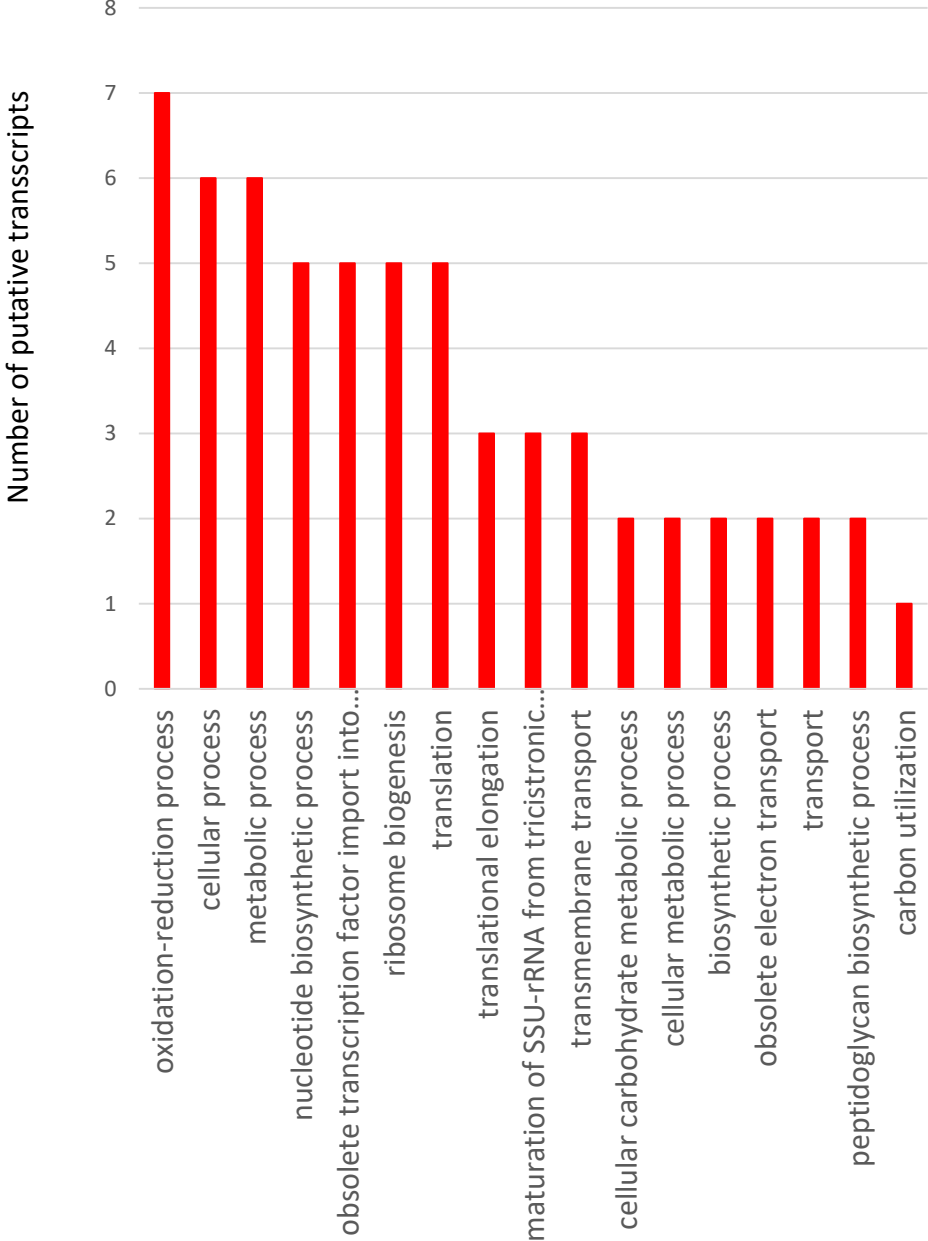

C. Molecular function

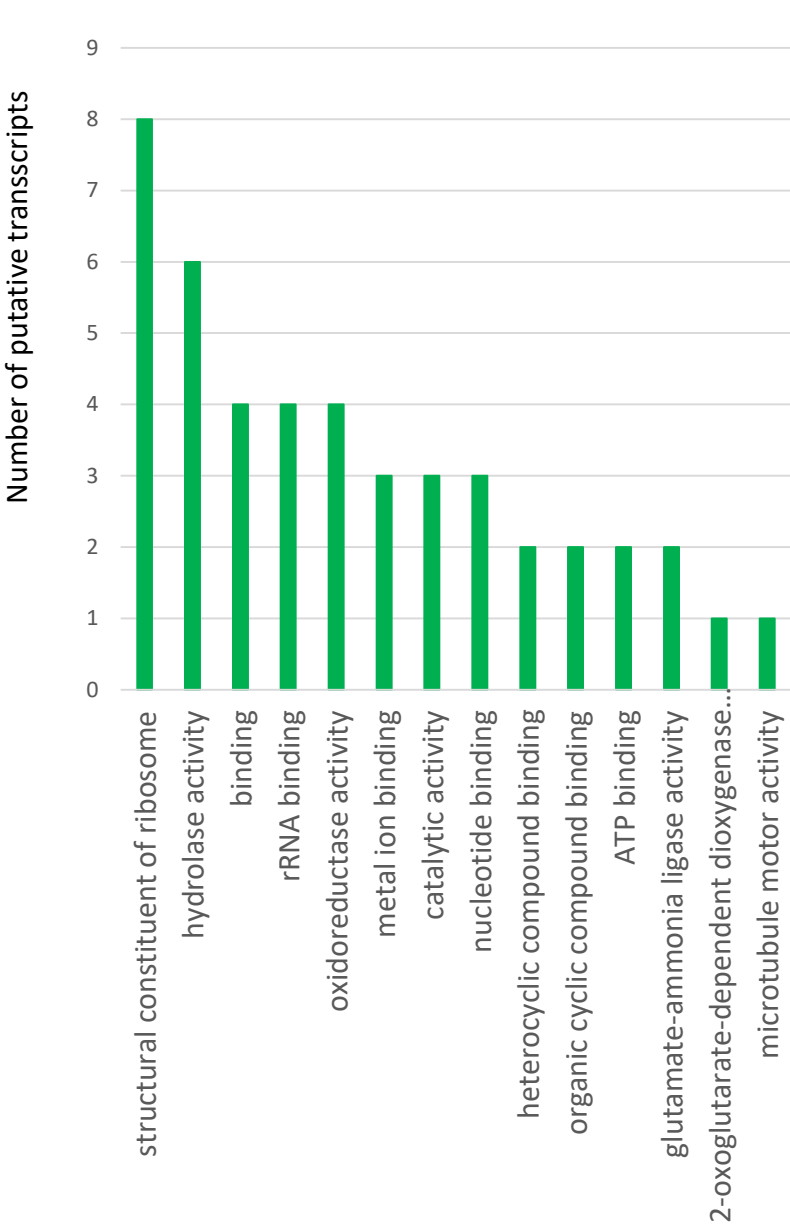

Supplement: Supplementary file 1 [file marinedrugs-18-00386-s001.zip › Supplementary files/Supplementary figure 1 go distribution.pdf]

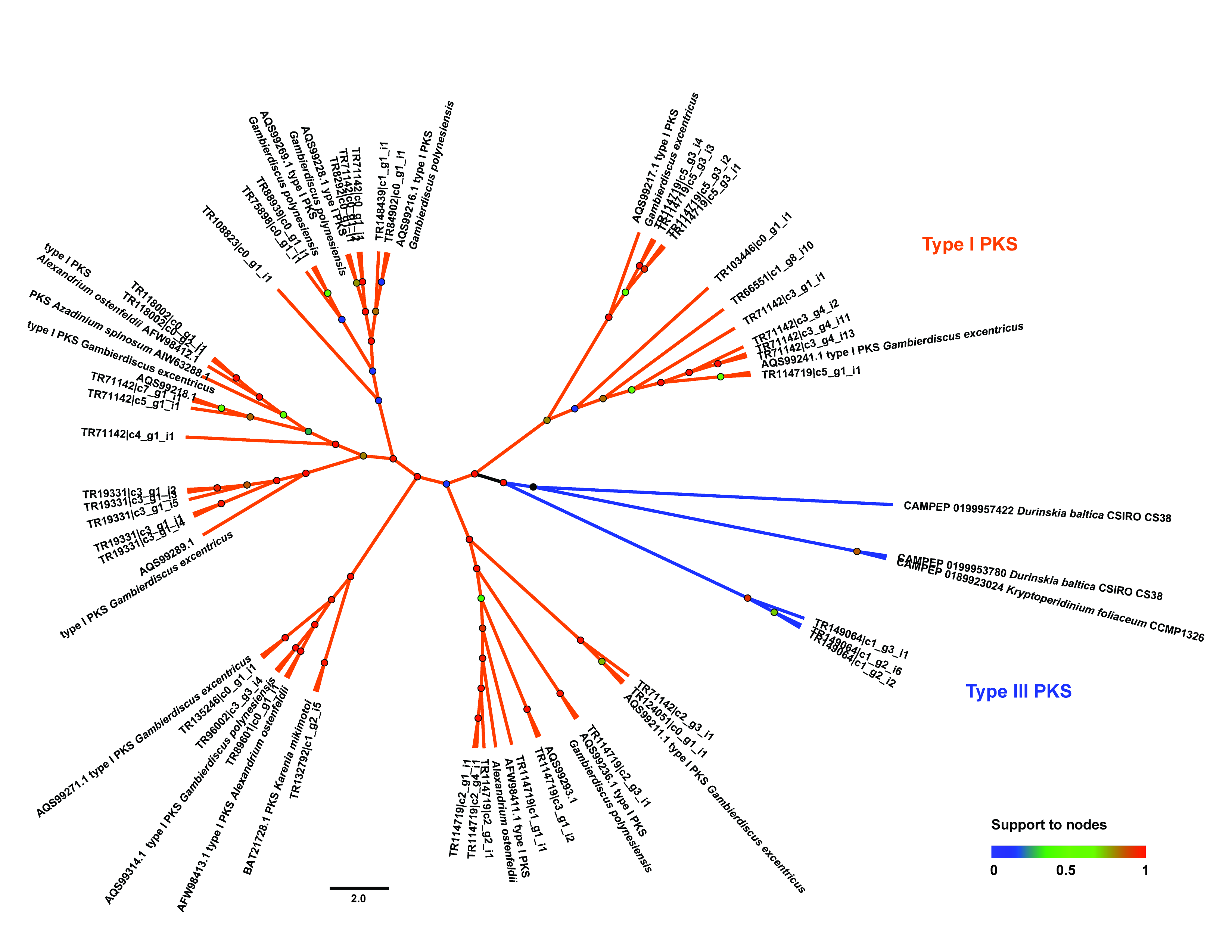

Supplement: Supplementary file 1 [file marinedrugs-18-00386-s001.zip › Supplementary files/Supplementary Figure 2.tif]

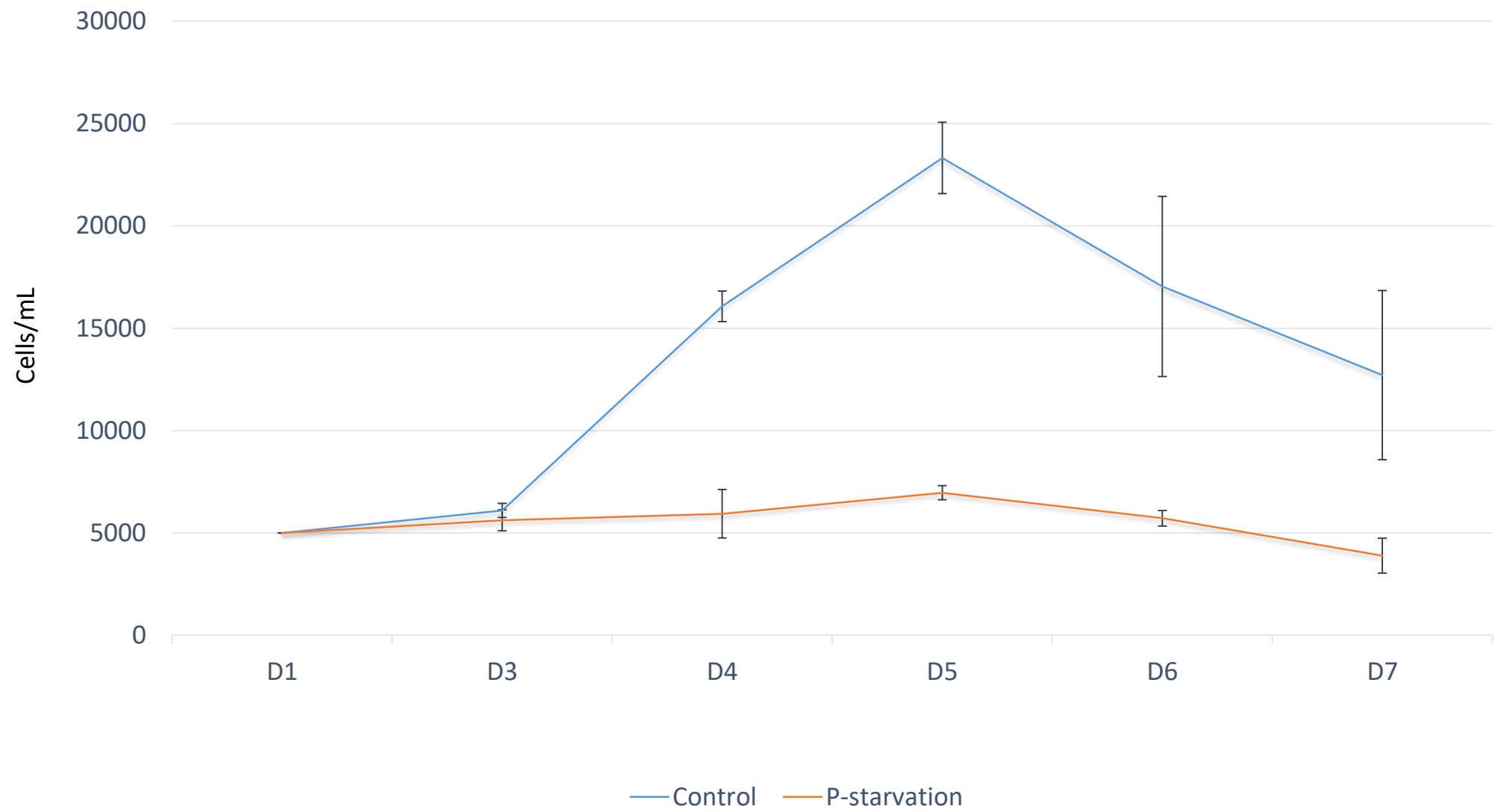

Supplement: Supplementary file 1 [file marinedrugs-18-00386-s001.zip › Supplementary files/Supplementary figure 3 growth curve.pdf]
